# Impact of dexamethasone use to prevent from severe COVID-19-induced acute kidney injury

**DOI:** 10.1186/s13054-021-03666-7

**Published:** 2021-07-16

**Authors:** Arthur Orieux, Pierre Khan, Renaud Prevel, Didier Gruson, Sébastien Rubin, Alexandre Boyer

**Affiliations:** 1 Service de Médecine Intensive Réanimation, Hôpital Pellegrin, Place Amélie Raba Léon, CHU de Bordeaux, 33000 Bordeaux, France; 2 Unité INSERM U1045, Université de Bordeaux, Bordeaux, France; 3 Service de Néphrologie, Transplantation, Dialyse, Aphérèses, Hôpital Pellegrin, CHU de Bordeaux, Bordeaux, France; 4 Unité INSERM U1034, Université de Bordeaux, Bordeaux, France

**Keywords:** Acute kidney injury, COVID-19, Dexamethasone, Intensive care unit

## To the editor

Since the first wave of COVID-19, the management of patients with severe COVID-19 in intensive care unit (ICU) has changed with the widespread use of dexamethasone (DXM) in severe patients [1]. Indeed, RECOVERY trial [2] showed a decrease in both mortality at day 28 and the number of patients who received renal replacement therapy (RRT) with DXM. We have previously reported a higher than usual incidence of acute kidney injury (AKI) (80%) in severe COVID-19 patients treated in ICU [3]. Like lung injury, specific SARS-CoV-2 inflammatory process was previously suggested in AKI pathogenesis [4, 5] and is susceptible to be improved by DXM. Besides, in another report, we recently described a 15% incidence of chronic kidney disease (CKD) at 3 months after COVID-19-induced AKI [6], all of these patients developing acute kidney disease (AKD) before CKD. The aim of this report was to assess the independent clinical effect of DXM on the prevention of severe COVID-19-induced AKI.

We carried out a prospective study in the medical ICU of the University Hospital of Bordeaux (France) from March 17, 2020, to December 20, 2020. All patients admitted for a severe COVID-19 were included. DXM was exclusively used during the second wave starting in Bordeaux (France) on August 6 at 6 mg per day for 10 days at ICU admission. Patients who presented AKI at ICU admission were excluded from the analysis.

## Patients characteristics (Table 1)

From March 17, 2020, 126 patients were admitted in our unit, 26/126 patients (21%) were excluded because of AKI at ICU admission (5 and 21 patients, in first and second waves, respectively). DXM was used in all patients in the second wave and in none of the first wave. In the 100 patients included, median age was 63 ± 11 years, 59/100 (59%) had hypertension, 10/100 (10%) suffered from CKD and 48/100 (48%) required mechanical ventilation (MV) in the first 24 h. In the study population, 56/100 (56%) patients developed AKI: 39/52 (75%) and 17/48 (35%) patients (*p* < 0.001), in the first and second waves, respectively. AKD was observed in 24/100 (24%) of patients: 14/52 (27%) patients in the first wave and 10/48 (21%) in the second wave (*p* = 0.49).

**Table 1:** Comparison of baseline characteristics and outcomes

	Total *n* = 100	First wave *n* = 52	Second wave *n* = 48	*p*-value
*Baseline characteristics*				
Males, No. (%)	76 (76)	38 (73)	38 (79)	0.49
Age (years), mean (SD)	63 ± 11	61 ± 12	66 ± 10	0.009
BMI, mean (SD)	30 ± 5	31 ± 4	30 ± 5	0.20
CKD, No. (%)	10 (10)	4 (8)	6 (12)	0.51
Hypertension, No. (%)	59 (59)	30 (58)	29 (60)	0.84
Diabetes, No. (%)	33 (33)	15 (29)	18 (37)	0.40
Ischemic cardiopathy, No. (%)	13 (13)	4 (8)	9 (19)	0.14
Chronic respiratory disease, No. (%)	17 (17)	7 (13)	10 (21)	0.43
Immunosuppression, No. (%)	9 (9)	4 (8)	5 (10)	0.73
RASi exposure, No. (%)	35 (35)	18 (35)	17 (36)	1
Time between first symptoms and ICU admission (days), mean (SD)	8 ± 4	8 ± 4	8 ± 3	0.74
Time between first symptoms and hospital admission (days), mean (SD)	7 ± 3	7 ± 3	6 ± 4	0.49
Time between hospital admission and ICU admission (days), mean (SD)	1 ± 2	1 ± 2	2 ± 3	0.31
*In the first 24 h of ICU hospitalization (before AKI development)*				
SAPS II, median [IQR]	40 [30–52]	40 [25–60]	40 [33–46]	0.65
Catecholamine use, No. (%)	9 (9)	8 (15)	1 (2)	0.03
Mechanical ventilation, No. (%)	48 (48)	39 (75)	9 (19)	< 0.001
Worst PaO_2_/FiO_2_ ratio, median [IQR]	146 [98–192]	146 [89–205]	146 [116–181]	0.76
Intravenous fluid therapy (L), median [IQR]	2.36 [1.62–3.15]	2.77 [2.13–3.63]	1.94 [1.42–2.70]	0.0023
Dexamethasone use, No. (%)	48 (48)	0 (0)	48 (100)	1
*Main outcomes*				
ICU mortality, No. (%)	19 (19)	7 (13)	12 (25)	0.20
ICU length (days), median [IQR]	12 [6–25]	13 [7–27]	11 [5–22]	0.26
Hospitalization length (days), median [IQR]	26 [14–39]	27 [17–45]	23 [13–30]	0.04
Mortality at D28, No. (%)	21 (21)	9 (17)	12 (25)	0.46
*Renal outcomes*				
AKI, No. (%)	56 (56)	39 (75)	17 (35)	< 0.001
*AKI stage*				0.24
KDIGO 1, No. (%)	19 (34)	13 (33)	6 (35)	
KDIGO 2, No. (%)	18 (32)	15 (38)	3 (18)	
KDIGO 3, No. (%)	19 (34)	11 (28)	8 (47)	
Renal replacement therapy, No. (%)	12 (12)	7 (13)	5 (10)	0.76
Time between AKI and ICU admission (days), median [IQR]	4 [2–8]	4 [3–7]	4 [1–9]	0.74
AKD, No. (%)	24 (24)	14 (27)	10 (21)	0.49

Chronic respiratory disease includes chronic obstructive bronchopneumonia and asthma. The Kidney Disease: Improving Global Outcomes (KDIGO) definition was used to define AKI Stages 1, 2 and 3. Serum creatinine (SCr) and urine output were both taken into account. Continuous variables are reported as mean (standard deviation) or median [interquartile range] if the variable did not fit a normal distribution and categorical variables are reported as numbers (percentages). Quantitative variables were compared using a *t*-test, and qualitative variables were compared using Fisher’s exact test

AKD, acute kidney disease; AKI, acute kidney injury; BMI, body mass index; CKD, chronic kidney disease; D28, day 28; KDIGO, Kidney Disease Improving Global Outcomes; ICU, intensive care unit; IQR, interquartile; SAPSII, Simplified Acute Physiology Score; SCr, serum creatinine; SD, standard deviation; RASi, renin–angiotensin system inhibitor

In univariate analysis, the use of DXM (OR = 0.18 [0.07–0.42]), hypertension (OR = 2.31 [1.03–5.29]), SAPSII (OR = 1.04 [1.01–1.07]), MV (OR = 8.92 [3.65–23.66]) and intravenous fluid therapy (OR = 1.67 [1.20–2.47]) were significantly associated with AKI. In multivariate analysis, MV (OR = 5.02 [1.68–15.78]) and DXM use (OR = 0.31 [0.09–0.99]) remained significantly associated with AKI.

## Risk factors for AKI (Table 2)

**Table 2:** Univariate and multivariate analysis for AKI development

	Univariate analysis		Multivariate analysis	
	OR (AKI)	CI95%	OR (AKI)	CI 95%
*Baseline characteristics*				
Males, No. (%)	0.88	[0.34–2.22]		
Age (years), mean (SD)	1.006	[0.97–1.04]	1.02	[0.97–1.07]
BMI, mean (SD)	1.05	[0.98–1.14]		
CKD, No. (%)	3.50	[0.82–24.05]	4.10	[0.71–33.79]
Hypertension, No. (%)	2.31	[1.03–5.29]	1.93	[0.66–5.78]
Diabetes, No. (%)	0.76	[0.33–1.77]		
Ischemic cardiopathy, No. (%)	1.91	[0.58–7.50]		
Chronic respiratory disease, No. (%)	1.15	[0.40–3.44]		
Immunosuppression, No. (%)	1.64	[0.41–8.14]		
RASi exposure, No. (%)	1.54	[0.67–3.64]		
Time between first symptoms and ICU admission (days), mean (SD)	0.97	[0.87–1.07]		
*In the first 24 h of ICU hospitalization (before AKI development)*				
SAPS II, median [IQR]	1.04	[1.01–1.07]		
Catecholamine use, No. (%)	3	[0.68–20.87]		
Mechanical ventilation, No. (%)	8.92	[3.65–23.66]	5.02	[1.68–15.78]
Worst PaO_2_/FiO_2_ ratio, median [IQR]	0.99	[0.98–1]		
Intravenous fluid therapy (L), median [IQR]	1.67	[1.20–2.47]	1.35	[0.90–2.14]
Dexamethasone use, No. (%)	0.18	[0.07–0.42]	0.31	[0.09–0.99]

Univariate and multivariate analysis was proceeded using logistic regression. To choose independent variables included in the multivariate model, we allowed one independent variable for every 10 dependent variable’s outcomes analyzed. Independent variables with a *p*-value < 0.20 in the univariate analysis were taken into consideration, and we kept variables which were previously described to be associated with AKI or highly associated (*p* < 0.05) with AKI in the univariate analysis. Absence of collinearity was checked

AKI, acute kidney injury; BMI, body mass index; CKD, chronic kidney disease; ICU, intensive care unit; IQR, interquartile; SAPSII, Simplified Acute Physiology Score; SD, standard deviation; RASi, renin–angiotensin system inhibitor

## Risk factors for AKD

In univariate analysis, prior CKD (OR = 13.8 [3.14–97.44]), chronic respiratory disease (OR = 5.16 [1.54–19.19]), immunosuppression (OR = 7.67 [1.60–55.75]) were significantly associated with AKD. In multivariate analysis, only prior CKD (OR = 17.73 [2.91–108.08]) was significantly associated with AKD (DXM use, OR = 1.10 [0.32–3.81]).

Our study suggests an independent effect of DXM to prevent from AKI in severe COVID-19 patients. This result needs to be confirmed in larger studies.

High rate of vasopressors and greater intravenous fluid therapy reported during the first wave imaged the earlier and more frequent use of MV, without reflecting the patients' severity admitted in our ICU. Indeed, patients admitted during the second wave had more comorbidities and were older, which explains the increased mortality trend observed in our study.

Lack of effect of DXM on RRT in our study can be explained by a lack of power and by the too weak effect of corticosteroids on AKI.

Our results could indicate an important impact of inflammatory state in the pathogenesis of COVID-19-induced AKI. These results support the hypothesis that corticosteroid therapy can reduce “inflammatory” AKI incidence (specific of COVID-19 infection) but has no impact on "maladaptive repair" lesions secondary to AKI that can lead to an AKD.

## Data Availability

The dataset used and analyzed for the current study is available from the corresponding author on reasonable request.
